# Evidence of nerve agent VX exposure in rat plasma by detection of albumin-adducts in vitro and in vivo

**DOI:** 10.1007/s00204-023-03521-4

**Published:** 2023-06-01

**Authors:** Tamara Kranawetvogl, Andreas Kranawetvogl, Lisa Scheidegger, Timo Wille, Dirk Steinritz, Franz Worek, Horst Thiermann, Harald John

**Affiliations:** 1 Bundeswehr Institute of Pharmacology and Toxicology, Neuherbergstr. 11, 80937 Munich, Germany; 2 Walther-Straub-Institut, Ludwig-Maximilians-Universität, Munich, Germany; 3 Central Institute of the Bundeswehr Medical Service Munich, Garching, Germany

**Keywords:** Verification, Albumin, Dipeptide, Disulfide-adduct, Forensic, Nerve agent, Cysteine

## Abstract

VX is a highly toxic organophosphorus nerve agent that reacts with a variety of endogenous proteins such as serum albumin under formation of adducts that can be targeted by analytical methods for biomedical verification of exposure. Albumin is phosphonylated by the ethyl methylphosphonic acid moiety (*EMP*) of VX at various tyrosine residues. Additionally, the released leaving group of VX, 2-(diisopropylamino)ethanethiol (*DPAET*), may react with cysteine residues in diverse proteins. We developed and validated a microbore liquid chromatography-electrospray ionization high-resolution tandem mass spectrometry (µLC-ESI MS/HR MS) method enabling simultaneous detection of three albumin-derived biomarkers for the analysis of rat plasma. After pronase-catalyzed cleavage of rat plasma proteins single phosphonylated tyrosine residues (Tyr-*EMP*), the Cys^34^(-*DPAET*)Pro dipeptide as well as the rat-specific LeuProCys^448^(-*DPAET*) tripeptide were obtained. The time-dependent adduct formation in rat plasma was investigated in vitro and biomarker formation during proteolysis was optimized. Biomarkers were shown to be stable for a minimum of four freeze-and-thaw cycles and for at least 24 h in the autosampler at 15 °C thus making the adducts highly suited for bioanalysis. Cys^34^(-*DPAET*)Pro was superior compared to the other serum biomarkers considering the limit of identification and stability in plasma at 37 °C. For the first time, Cys^34^(-*DPAET*)Pro was detected in in vivo specimens showing a time-dependent concentration increase after subcutaneous exposure of rats underlining the benefit of the dipeptide disulfide biomarker for sensitive analysis.

## Introduction

Organophosphorus nerve agents (OPNA) are chemical warfare agents (CWA) banned by the Chemical Weapons Convention (CWC) since 1997 prohibiting their development, production, stockpiling and transport as well as their deployment (Organisation for the prohibition of chemical weapons 2020a). Adherence to the CWC is supervised by the Organisation for the Prohibition of Chemical Weapons (OPCW) coordinating an international network of specialized designated laboratories (Organisation for the prohibition of chemical weapons 2020a). OPNA still represent a considerable threat and have been used repeatedly in terroristic attacks (Nagao et al. 1997; Ohbu et al. 1997), assassinations (Nakagawa and Tu 2018), attempted murder (Organisation for the prohibition of chemical weapons 2018; Steindl et al. 2021) and during the ongoing Syrian Arab Republic conflict (General assembly security council 2013; John et al. 2018b). They are characterized by high acute toxicity due to the inhibition of acetylcholinesterase (AChE) which induces a cholinergic crisis that might ultimately lead to death by central respiratory depression and peripheral respiratory muscle dysfunction (Grob and Harvey 1953; Munro 1994). Accordingly, reliable forensic methods are of essential relevance for unambiguous verification of an alleged use of OPNA.

**Fig. 1:**
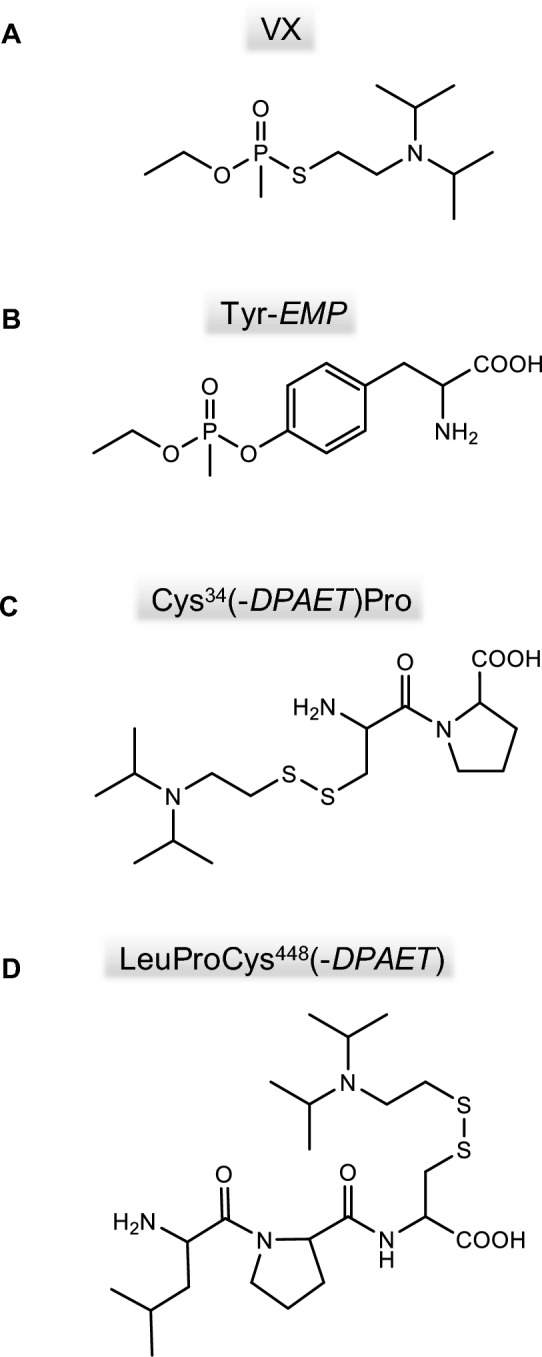
Structures of the nerve agent VX and the rat serum albumin-derived biomarkers. **A** Nerve agent VX; **B** tyrosine residue phosphonylated by the VX-derived ethyl methylphosphonic acid, Tyr-*EMP*, **C** dipeptide Cys^34^Pro adducted by the leaving group of VX 2-(diisopropylamino)ethanethiol, Cys^34^(-*DPAET*)Pro, **D** tripeptide LeuProCys^448^ adducted by the leaving group, LeuProCys^448^(-*DPAET*). Rat serum albumin-derived biomarkers were obtained after proteolysis of VX-exposed plasma with pronase

Gas and liquid chromatography (LC) coupled to mass spectrometry (MS) are commonly used for biomedical verification of OPNA exposure in humans and animals, either targeting the residual poison, their hydrolysis products or their reaction products with endogenous proteins (adducts) (Black and Read 2013; John et al. 2008, 2020, 2021; Noort et al. 2002). Adducts of OPNA with proteins such as albumin, AChE and butyrylcholinesterase (BChE) are stable in plasma for several weeks and suited for retrospective post-exposure analysis (Dafferner et al. 2017; Fidder et al. 2002; John et al. 2020, 2021; Lee et al. 2018; Peeples et al. 2005). Following exposure to the OPNA VX (Fig. 1A) albumin is phosphonylated by the ethyl methylphosphonic acid-moiety (*EMP*) at diverse tyrosine residues, especially at Tyr^411^ (John et al. 2010, 2021; Li et al. 2007; Noort et al. 2009) as well as at serine and lysine residues (Ding et al. 2008; Fu et al. 2019a; John et al. 2020). The single amino acid adduct Tyr-*EMP* (Fig. 1B; John et al. 2010, 2021; Li et al. 2007; Noort et al. 2009; von der Wellen et al. 2018) is generated by subjecting VX-exposed plasma proteins to proteolysis with pronase representing a valuable biomarker of exposure in vitro and in vivo (Bao et al. 2012; Kranawetvogl et al. 2016, 2017, 2018a; Lee et al. 2018; Williams et al. 2007). In addition, disulfide- adducts between the thiol-containing leaving group of VX, 2-(diisopropylamino)ethanethiol (*DPAET*), and the free thiol group of Cys^34^ are liberated as Cys^34^(-*DPAET*)Pro after proteolysis with pronase (Fig. 1C; Kranawetvogl et al. 2016, 2017, 2018a). Tyr-*EMP* and Cys^34^(-*DPAET*)Pro were analyzed simultaneously by microbore liquid chromatography-electrospray ionization high-resolution tandem-mass spectrometry (µLC-ESI MS/HR MS) operating in the product ion scan mode (PIS) (Kranawetvogl et al. 2016, 2017, 2018a). Accordingly, the structure of both, the phosphonyl moiety of the OPNA as well as its leaving group, were identified (Kranawetvogl et al. 2016, 2017, 2018a). Quite similarly, disulfide leaving-group adducts of certain pesticides like oxydemeton-*S*-methyl, dimethoate and omethoate have already proven their suitability in human samples for unequivocal identification of the incorporated poison (John et al. 2018a; Kranawetvogl et al. 2018b). Nevertheless, so far in vivo data of V-type OPNA adducts are missing not only for humans but also for any animal species. Therefore, adducts with rat plasma proteins may represent valuable analytical targets and broaden the toolbox for verification analysis. Especially rodents like rats may be of interest to investigate real case exposure scenarios as they are likely to be exposed to OPNA in rural and urban areas. The concentration of rat serum albumin (RSA, UniProtAcc. No. P02770) in plasma is similar to that in humans (40 mg/mL) (Metz und Schütze 1975) and RSA also contains a reactive Tyr^411^ and a Cys^34^ residue exhibiting a free thiol group (Chen et al. 2013) as well as a LeuProCys^448^ sequence as an analogue to the human MetProCys^448^ (Kranawetvogl et al. 2018a). Accordingly, we developed and validated a µLC-ESI MS/HR MS method for the qualitative detection of adducted biomarkers and applied it to an in vivo study of VX-exposed rats (Stigler et al. 2022).

## Materials and methods

### Chemicals

Water (LC–MS grade), acetonitrile (ACN, hypergrade for LC–MS) and formic acid (FA, ≥ 98%) were from Merck (Darmstadt, Germany), ammonium hydrogen carbonate (NH_4_HCO_3_, ultra grade, ≥ 99.5%) from Sigma-Aldrich (Steinheim, Germany), lithium-heparinized plasma from Wistar Hannover rats for VX in vitro exposure from NeoBiotech (Nanterre, France), pronase (EC 3.4.24.4) from *Streptomyces griseus* was from Roche (Mannheim, Germany) and deuterated atropine (*d*_*3*_-Atr) from CDN Isotopes (Pointe-Claire, Canada). A solution of *d*_*3*_-Atr (6 ng/mL in 0.5% v/v FA) was used as internal standard (IS). VX (CAS no. 50782-69-9) was made available by the German Ministry of Defense and tested for integrity in house by nuclear magnetic resonance (NMR) spectroscopy. A stock solution of VX (0.1% v/v, 1 mg/mL) and diverse working solutions with concentrations between 0.046 µg/mL and 0.38 mg/mL were prepared in ACN.

### Incubation of rat plasma with VX in vitro

Rat heparin plasma (240 µL) was mixed in vitro with VX working solutions (10 µL) followed by a 2 h incubation at 37 °C (standard conditions) under gentle shaking. The reference was made with a final VX concentration of 3.78 µg/mL if not stated otherwise. Blanks did not contain VX but ACN only. Standards were produced with final VX concentrations of 15.11 µg/mL, 7.55 µg/mL, 3.78 µg/mL, 1.89 µg/mL, 0.94 µg/mL, 0.47 µg/mL, 0.24 µg/mL, 0.12 µg/mL, 59.02 ng/mL, 29.51 ng/mL, 14.75 ng/mL, 11.07 ng/mL, 7.38 ng/mL, 5.54 ng/mL, 3.69 ng/mL, 2.77 ng/mL and 1.84 ng/mL. During method optimization incubation times were varied between 1 min and 10 d.

### Plasma sample preparation

According to the standard protocol rat plasma (50 µL) was mixed with ACN (100 µL) for protein precipitation, followed by centrifugation (10 min, 10,270 RCF, 15 °C), removal of the supernatant (supernatant I), washing the pellet two-times with ACN (100 µL, each) and drying it under a gentle stream of nitrogen. The dried pellet was re-suspended in pronase solution (100 µL, 12 mg/mL in 50 mM NH_4_HCO_3_) and incubated at 37 °C for 3 h under vigorous shaking. Afterwards, ACN (200 µL) was added prior to centrifugation (10 min, 10,270 RCF, 5 °C). An aliquot of the supernatant (200 µL) was evaporated to dryness under a gentle stream of nitrogen. The residue was dispersed under vigorous shaking in NH_4_HCO_3_ (60 µL, 50 mM) and *d*_*3*_-Atr solution (30 µL) followed by ultrasonication (10 min). After centrifugation (10 min, 10,270 RCF, 15 °C) the supernatant (80 µL, supernatant II) was analyzed by µLC-ESI MS/HR MS (method A, see below) to detect the biomarkers. For measurement of remaining VX in supernatant I an aliquot (5 µL) was evaporated to dryness, re-dissolved in *d*_*3*_-Atr solution (250 µL) and analyzed by µLC-ESI MS/HR MS (PIS) (method B, see below).

### µLC-ESI MS/HR MS (PIS) analysis

The µLC system consisted of a microLC 200 pump (Eksigent Technologies LLC, Dublin, CA, USA), an autosampler (HTC-xt DLW, CTC Analytics, Zwingen, Switzerland) with a sample tray kept at 15 °C and a 20 µL sample loop (Sunchrom, Friedrichsdorf, Germany). An Acquity UPLC® HSS T3 column (C18, 50 × 1.0 mm I.D., 1.8 µm, 100 Å, Waters, Eschborn, Germany) protected by a Security Guard™ Ultra Cartridge UHPLC precolumn (C18-peptide, 2.1 mm I.D., Phenomenex, Aschaffenburg, Germany) was used as stationary phase. Solvent A (0.05% FA) and solvent B (ACN/H_2_O 80:20 v/v, 0.05% FA) served as mobile phase in gradient mode with a flow of 30 µL/min. Chromatography was coupled to a hybrid quadrupole time-of-flight mass spectrometer (TT5600^+^, Sciex, Darmstadt, Germany) via an electrospray ionization (ESI, positive mode) interface. The mass spectrometer was operated in the PIS mode subjecting preselected protonated precursor ions to collision-induced dissociation (CID) using nitrogen as collision gas. Product ions were monitored in the range from *m/z* 50 to *m/z* 700 in high-resolution mode. Automated calibration was done by infusion of a calibration solution (APCI positive calibration solution, Sciex) after every fifth chromatographic run via an additional atmospheric pressure chemical ionization (APCI) inlet using a calibrant delivery system (Sciex). The following MS parameters were applied: ion source temperature 200 °C, ion spray voltage floating + 4500 V, ion source gas 1 40 psi (2.76 × 10^5^ Pa), ion source gas 2 50 psi (3.45 × 10^5^ Pa), curtain gas 30 psi (2.07 × 10^5^ Pa), declustering potential 60 V, collision energy spread 5 V, ion release delay 67 ms, ion release width 25 ms and accumulation time 150 ms. The entire µLC system was controlled by the Eksigent Control 4.3 and Analyst TF 1.8.1 software and MS data processing was done with PeakView 2.2.0 and Sciex OS 1.7 (all Sciex). The mentioned column, solvents, flow and general MS parameters were used for both method A and method B (see below).

### Biomarker analysis (method A)

Chromatography was carried out using the following gradient at 65 °C: t [min]/B [%]: 0/2, 12/39, 12.5/95, 14.5/95, 15/2, 20/2 with an initial 2 min equilibration period under starting conditions.

The two most intense qualifier product ions (Qual I and Qual II) of the protonated precursor ions ([M + H]^+^) of Tyr-*EMP* (*m/z* 288.1, 20 V) (Table 1), Cys^34^(-*DPAET*)Pro (*m/z* 378.2, 27.5 V) (Table 2) and LeuProCys^448^(-*DPAET*) (*m/z* 491.3, 30 V) (Table 3) and the IS (*m/z* 293.1, 42 V) were extracted from total ion chromatograms with an accuracy of ± 0.005 Th, each. Individual collision energy (CE) values were applied as listed in parenthesis.

**Table 1:**
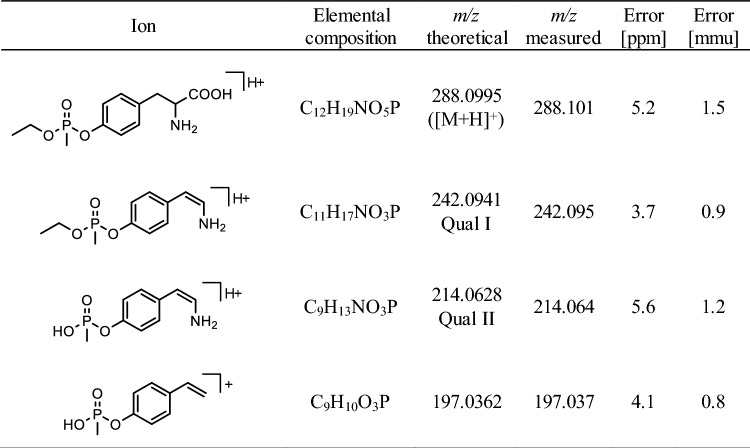
Precursor and product ion masses of protonated Tyr-*EMP*

*EMP*: VX-derived ethyl methylphosphonic acid, *Qual* qualifying ion, *[M + H]*^*+*^ singly protonated precursor ion

Collision-induced dissociation (CID) was carried out with a collision energy (CE) of 20 V

**Table 2:**
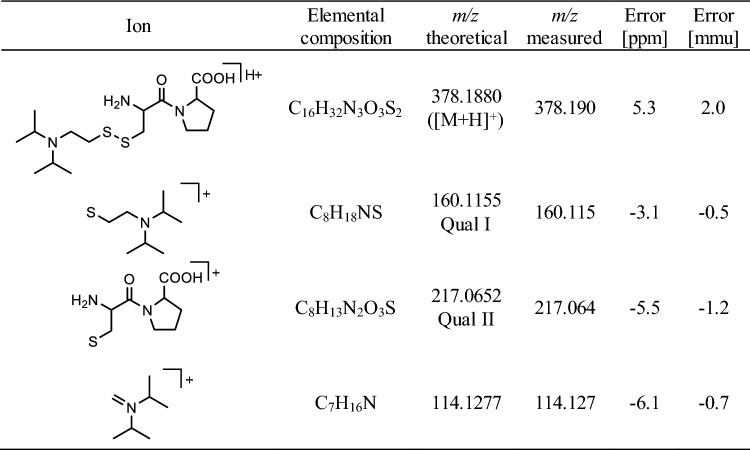
Precursor and product ion masses of protonated Cys^34^(-*DPAET*)Pro

*DPAET* 2-(diisopropylamino)ethanethiol, *Qual* qualifying ion, *[M + H]*^*+*^ singly protonated precursor ion

Collision-induced dissociation (CID) was carried out with a collision energy (CE) of 27.5 V

**Table 3:**
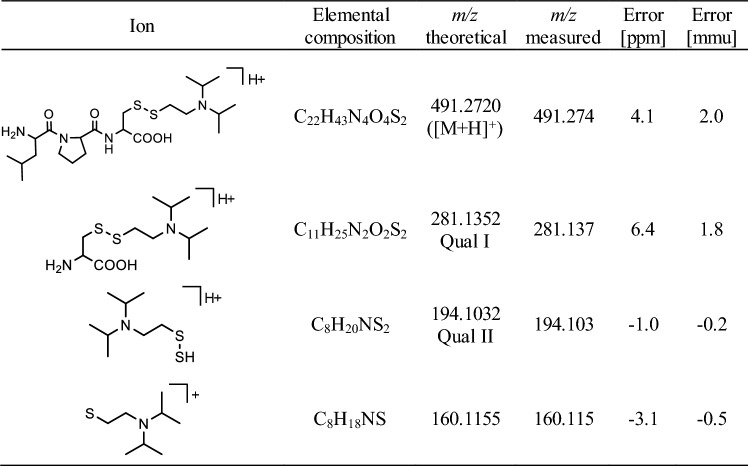
Precursor and product ion masses of protonated LeuProCys^448^(-*DPAET*)

*DPAET* 2-(diisopropylamino)ethanethiol, *Qual* qualifying ion, *[M + H]*^*+*^ singly protonated precursor ion

Collision-induced dissociation (CID) was carried out with a collision energy (CE) of 30.0 V

### VX analysis (method B)

The following gradient was applied at 60 °C: t [min]/B [%]: 0/10, 11/60, 11.5/95, 13.5/95, 14/10, 15/10 with an initial 5 min equilibration under starting conditions. Qual I and Qual II of protonated VX (*m/z* 268.1, 35 V) (Table 4) and the IS (see method A) were monitored after CID with the given CE values.

**Table 4:**
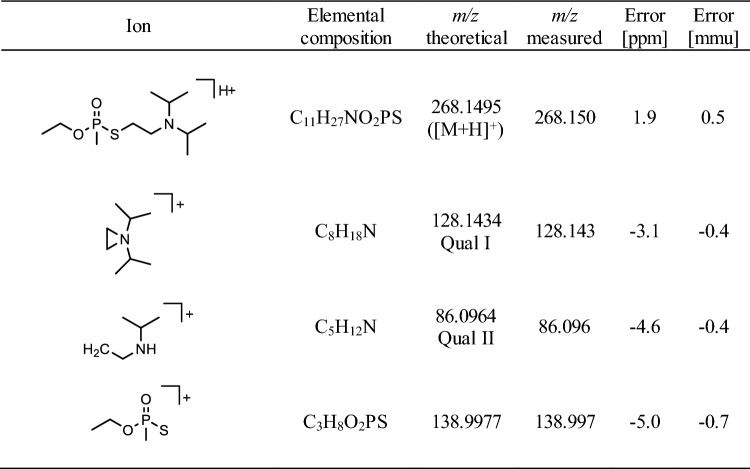
Precursor and product ion masses of protonated nerve agent VX

*Qual* qualifying ion, *VX* O-ethyl-S-(2-diisopropylaminoethyl)methylphosphonothioate, *[M + H]*^*+*^ singly protonated precursor ion

Collision-induced dissociation (CID) was carried out with a collision energy (CE) of 35 V

### Time-dependent adduct formation during incubation of rat plasma in vitro

Rat plasma was mixed with VX (final concentration 3,78 µg/mL) and incubated for 48 h at 37 °C (*n* = 2) to take aliquots (50 µL) after 0.017 h, 0.17 h, 0.5 h, 1 h, 2 h, 3 h, 4 h, 6 h, 8 h, 10 h, 24 h, 26 h, 28 h, 30 h, 32 h and 48 h followed by immediate protein precipitation. The washed protein pellets were stored at  – 20 °C before further sample preparation according to the standard protocol. Biomarkers were analyzed using method A and VX by method B. Resulting mean peak areas (M) and standard deviations (SD) of Qual I were plotted against the incubation time.

### Time-dependent formation of biomarkers during proteolysis

Plasma standard (750 µL, [VX] 15.11 µg/mL, *n* = 2) was precipitated with ACN (1500 µL). The protein pellet was washed two times (1500 µL ACN, each) and subsequently dried with nitrogen before the addition of pronase solution (1500 µL, 12 mg/mL in 50 mM NH_4_HCO_3_) for proteolysis. After 0 min, 2 min, 5 min, 8 min, 10 min, 15 min, 30 min, 45 min, 60 min, 75 min, 90 min, 105 min, 120 min, 150 min, 180 min, 210 min, 240 min, 270 min, 300 min and 360 min, aliquots (50 µL) were drawn and further processed according to the standard protocol with adjusted smaller volumes prior to analysis using µLC-ESI MS/HR MS (PIS) (method A). Resulting peak areas (M ± SD) of Qual I were plotted against the incubation time.

### Selectivity of biomarker detection

Blank plasma from six individual rats was analyzed with µLC-ESI MS/HR MS (PIS) (method A) for the presence of interferences for any biomarker.

### Dose–response analysis and limit of identification of biomarkers

Sixteen rat plasma standards covering VX concentrations from 1.84 ng/mL to 7.55 µg/mL (*n* = 3, each) were prepared according to the standard protocol and analyzed by µLC-ESI MS/HR MS (PIS) (method A). Resulting peak areas (M ± SD) of the biomarkers (Qual I) were plotted against the VX concentration to characterize the dose–response. The limit of identification (LOI) was defined as the lowest VX concentration that still allowed biomarker detection in all three replicates and still met the peak area ratio of Qual II/Qual I determined from a reference within a given tolerance interval (Organisation for the prohibition of chemical weapons 2020b).

### Stability of biomarkers in the autosampler

Ten references ([VX] 7.55 µg/mL) were prepared according to the standard protocol and final supernatants (supernatant II) ready for analysis were pooled. This solution was stored in the autosampler for 24 h at 15 °C and analyzed every hour by µLC-ESI MS/HR MS (PIS) (method A). Relative biomarker concentrations were measured by their peak areas (Qual I).

### Freeze-and-thaw stability of protein adducts in plasma

References were analyzed (method A) to detect biomarkers at day 0 (24 h at 37 °C after spiking of VX) and after four freeze-and-thaw cycles (day 1, 2 and 3) in triplicate, each. Each cycle comprised freezing and storage for at least 24 h at  – 20 °C followed by thawing and storage for 1 h at room temperature. M ± SD of biomarker peak areas (Qual I) were determined to monitor the stability of relevant protein adducts.

### Adduct stability in plasma at 37 °C

References (*n* = 3) were stored for 10 d at 37 °C under gentle shaking to measure biomarkers at indicated time points in triplicate (method A) after sample preparation following the standard protocol: 2 h, 6 h, 8 h, 24 h, 32 h, 48 h, 56 h, 72 h, 96 h, 168 h, 192 h, 216 h and 240 h. M ± SD of biomarker peak areas (Qual I) were determined to follow the relative concentration as a measure of the relevant protein adduct amount.

### Application to in vivo samples

The µLC-ESI MS/HR MS (PIS) method (method A) for biomarker detection was applied to heparin plasma obtained from an in vivo study with male Wistar rats. Details on the scope, conditions and results of the study are described by Stigler et al. (Stigler et al. 2022). In brief, male Wistar rats were anaesthetized under continuous surveillance of anaesthesia depth and exposed to VX subcutaneously (s.c.) using a dose of 25 µg/kg (approximately 2 × LD_50_ for rats *i.m.)* (Bajgar 2005). Blood was drawn from the *arteria carotis* 1 min before and 3 min, 6 min, 10 min, 15 min, 30 min, 45 min and 60 min after challenge with VX. Following centrifugation heparinized plasma was stored at -20 °C prior to sample preparation. The animal study was in accordance with the German Animal Welfare Act (BGBI. I S. 1206, 1313; May 18th, 2006) and the European Parliament and Council Directive 2010/63/EU (September 22nd, 2010) under conditions minimizing animal suffering. Approval for the entire experimental procedure was given by the institutional animal protection committee (Ref.-No. 42-34-30-40/G03-19).

### Safety considerations

VX is a highly toxic nerve agent that must be handled by trained personnel wearing laboratory protective clothes. Working under the fume hood in specially equipped facilities and decontamination of materials that had contact to VX is strictly required. Decontamination should be done by dunking the material into alkaline NaOCl solution and storing it for several hours.

## Results and discussion

**Fig. 2:**
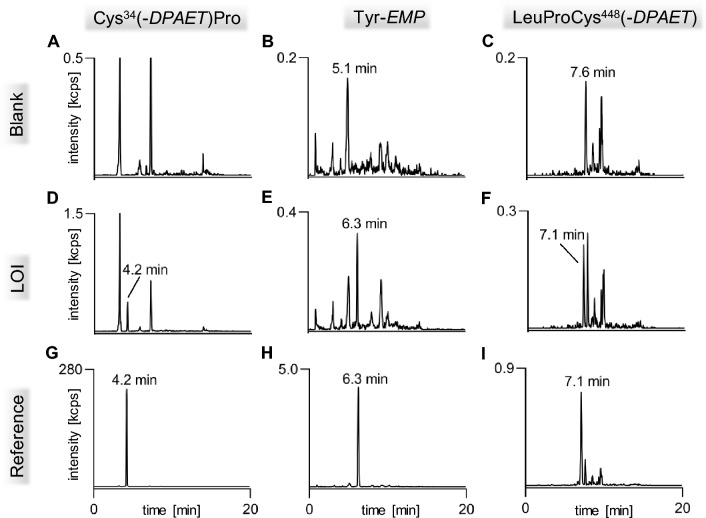
Detection of rat serum albumin-derived biomarkers. Extracted ion chromatograms (XIC) of the adducts from micro liquid chromatography-electrospray ionization high-resolution tandem mass spectrometry (µLC-ESI MS/HR MS) analysis; first column: screening for Cys^34^*(-DPAET)*Pro in **A** blank rat plasma, **D** a rat plasma standard spiked with VX at the limit of identification (LOI) level, and **G** a VX rat plasma reference; second column: screening for Tyr-*EMP* B) blank, **E** standard at LOI, **H** reference; third column: screening for LeuProCys^448^(-*DPAET*) **C** blank, **F** standard at LOI, **I** reference. For clarity reasons only the traces of the individual most intense qualifying ions (Qual I ± 0.005 Th, Tables 1, 2, 3) are shown. Cys^34^*(-DPAET)*Pro: Cys^34^Pro adducted by the leaving group of VX 2-(diisopropylamino)ethanethiol; LeuProCys^448^(-*DPAET*): LeuProCys^448^ adducted with DPAET; Tyr-*EMP*: tyrosine residue phosphonylated by the VX-derived ethyl methylphosphonic acid.

**Fig. 3:**
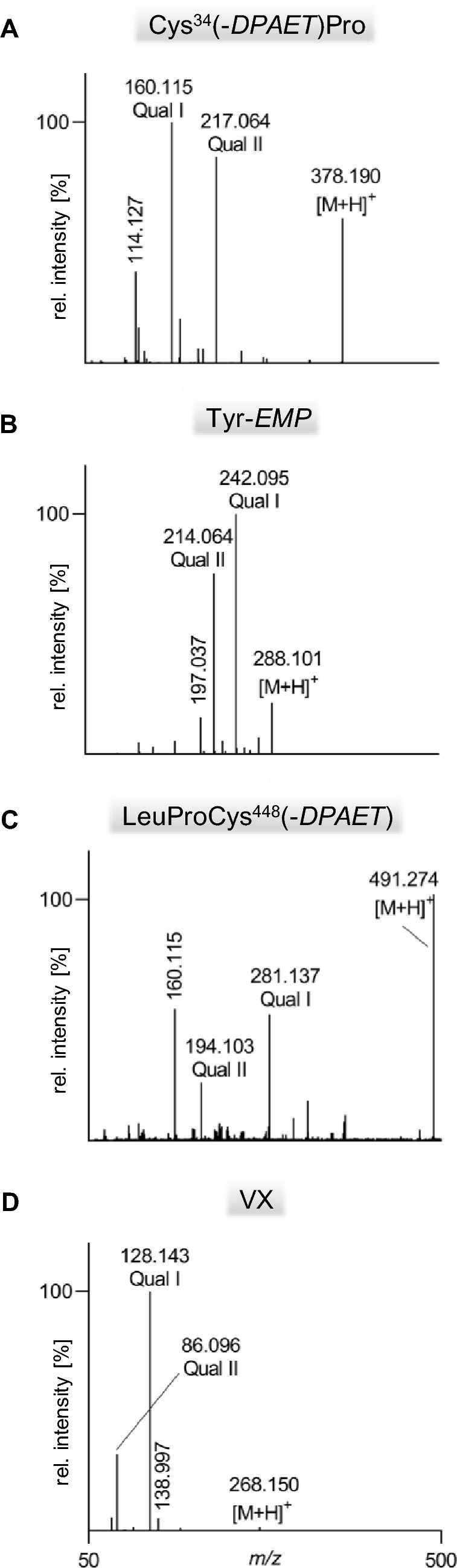
Product ion mass spectra of protonated rat serum albumin-derived biomarkers and VX. **A** Dipeptide Cys^34^Pro adducted by the leaving group of VX 2-(diisopropylamino)ethanethiol, Cys^34^(-*DPAET*)Pro, **B** tyrosine residue phosphonylated by the VX-derived ethyl methylphosphonic acid moiety, Tyr-*EMP*, **C** tripeptide LeuProCys^448^ adducted by the leaving group, LeuProCys^448^(-*DPAET*), **D** nerve agent VX. Spectra were extracted from µLC-ESI MS/HR MS analyses in product ion scan (PIS) mode of a rat plasma reference. The structural assignment of product ions are given in Tables 1, 2, 3, 4. Qual: qualifier ion; [M + H]^+^: single protonated precursor ion

Recently, we introduced methods for the simultaneous detection of V-agent adducts including phosphonylated Tyr residues as well as disulfide-adducts between Cys and the agent´s leaving group containing a thiol-group (Kranawetvogl et al. 2016, 2017, 2018a). These methods were shown to be well-suited for unambiguous identification of in vitro exposure to VX, Chinese VX (CVX) and Russian VX (RVX). In addition, poisoning with pesticides possessing thiol-containing leaving groups (e.g., oxydemeton-methyl, dimethoate, omethoate) were proven by similar methods (John et al. 2018a; Kranawetvogl et al. 2018b). However, adduct formation of the disulfide-adducts in vivo and adaption of the methods to non-human species were still missing. Therefore, we decided to develop an improved µLC-ESI MS/HR MS (PIS) method to detect Tyr-*EMP* (Fig. 1B), Cys^34^*(-DPAET)*Pro (Fig. 1C) as well as LeuProCys^448^*(-DPAET)* (Fig. 1D) in rat plasma suited for the verification of VX (Fig. 1A) exposure. The latter tripeptide adduct is a rat-specific analogue of MetProCys^448^(*-DPAET*) found in human serum albumin (HSA) (Kranawetvogl et al. 2016). After in vitro incubation of rat plasma with VX we succeeded in the detection of these postulated three adducts after pronase-catalyzed proteolysis as illustrated in Fig. 2. The most polar biomarker Cys^34^(*-DPAET*)Pro eluted at retention time (t_R_) 4.2 min (Fig. 2G); Tyr*-EMP* at t_R_ 6.3 min (Fig. 2H) and the most hydrophobic adduct LeuProCys^448^(*-DPAET*) at t_R_ 7.1 min (Fig. 2I). Blanks were free of these peaks as exemplarily illustrated in Fig. 2A–C indicating the high selectivity of the method for rat plasma analysis. The corresponding product ion spectra of these adducts were extracted from the µLC-ESI MS/HR MS (PIS) run and are shown in Fig. 3A–-C. The structural assignments of the three most intense product ions of each analyte are listed in Table 1 (Tyr-*EMP*), Table 2 (Cys^34^(-*DPAET*)Pro), and Table 3 (LeuProCys^448^(-*DPAET*)). The mass accuracy of each ion representing the difference between the measured accurate mass and the theoretical exact mass was excellent (< 10 ppm) underlining the plausibility of structures.

### Time-dependent adduct formation during incubation of rat plasma in vitro

**Fig. 4:**
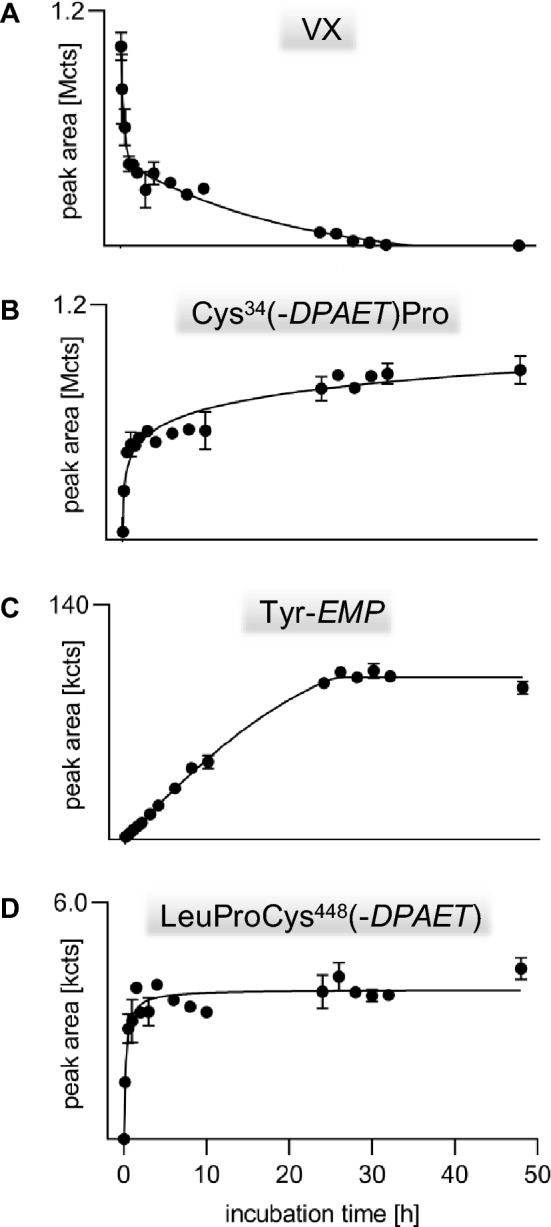
VX elimination and formation of biomarker-adducts during in vitro incubation of rat plasma with VX. **A** nerve agent VX, **B** dipeptide Cys^34^Pro adducted by the leaving group of VX 2-(diisopropylamino)ethanethiol, Cys^34^(-*DPAET*)Pro, **C** tyrosine residue phosphonylated by the VX-derived ethyl methylphosphonic acid, Tyr-*EMP*, **D** tripeptide LeuProCys^448^ adducted by the leaving group, LeuProCys^448^(-*DPAET*). Rat plasma was incubated with VX (3.78 µg/mL) for 48 h at 37 °C. At time points indicated remaining VX and biomarkers were analyzed by µLC-ESI MS/HR MS. Data points represent M ± SD of respective peak areas (qualifier ion Qual I, Tables 1, 2, 3, 4) obtained from triplicate incubations with VX.

Concentration–time profiles of the three adducts as well as of VX are depicted in Fig. 4. The identity of VX was confirmed by its MS/MS spectrum (Fig. 3D) and assignment of product ion signals to chemical structures (Table 4). VX (Fig. 4A) was rapidly degraded by about 60% in the initial 2 h period. Afterwards, the VX concentration decreased much slower showing complete degradation after 32 h. Such a biphasic elimination profile of VX was also described for human and rat plasma in vitro (Kranawetvogl et al 2018a; Reiter et al. 2011) as well as for swine and guinea pig plasma in vivo (van der Schans et al. 2003; Reiter et al. 2008, 2011) indicating similar processes of enantio-selective degradation*.* Elimination and degradation might have been due to (1) (non)-covalent interaction with highly abundant plasma proteins like albumin, (2) covalent reaction with enzymes like AChE, BChE or carboxylesterase (CXE) leading to inhibition of the enzyme, (3) enzymatic hydrolysis mediated by organophosphorus hydrolases (OPH) without inhibition of the enzyme itself or (4) non-enzymatic hydrolysis, i. e. spontaneous hydrolysis in aqueous media (Black 2010; John et al. 2020; Jokanovic et al. 1996; Jokanovic et al. 2001; Munro et al. 1999).

Due to the lack of a VX detoxifying OPH in rats and the high stability of VX the enzymatic and non-enzymatic hydrolysis was of minor (Black 2010; Bonierbale et al. 1997; John et al. 2020; Reiter et al. 2008), whereas the adduction to any plasma protein was of major relevance except of rat CXE characterized by poor reactivity (Maxwell 1992; Maxwell and Brecht 2001; van der Schans et al. 2003). The biphasic course with a rapid initial elimination of VX might be explained by a very fast reaction of VX with highly reactive targets leading to excessive release of the leaving group followed by a slower phase targeting lesser reactive targets.

Accordingly, the concentrations of Cys^34^*(-DPAET)*Pro (Fig. 4B) and LeuProCys^448^(*-DPAET*) (Fig. 4D) rapidly increased in this initial phase. In contrast, the Tyr*-EMP* concentrations rose linearly within the initial 24 h (Fig. 4C). This might be due to the existence of numerous targets prone to phosphonylation like highly reactive enzymes and other amino acid residues (Ding et al. 2008; Fu et al. 2019a, b) combined with varying reactivity of the different Tyr moieties.

A plateau was reached after 24 h (Fig. 4C) documenting that no further phosphonylation of Tyr residues in any protein occurred correlating to the nearly complete consumption of VX (Fig. 4A). The formation of the LeuProCys^448^*(-DPAET)* disulfide-adduct was only observed within the first 2 h reaching a stable plateau (Fig. 4D). This profile might have been due to the high excess of the free thiolate leaving group *DPAET* or its dimer tetraisopropylcystamine (Kranawetvogl et al. 2018a) produced during the initial phase of phosphonylation by VX allowing reaction with disulfide-bridged Cys^448^. Due to the low peak areas of this adduct, it is less suited as biomarker for verification analysis and was thus not evaluated in more detail. These results are in good accordance with findings in human plasma (Kranawetvogl et al. 2018a) thus proving good interspecies transferability of the applied method.

### Time-dependent formation of biomarkers during proteolysis

**Fig. 5:**
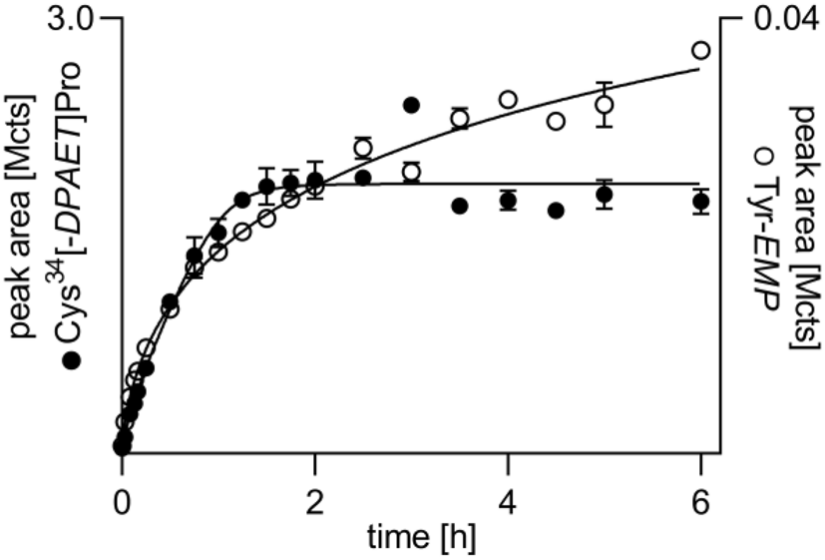
Formation of biomarker-adducts during proteolysis of VX-exposed rat plasma proteins. Rat heparin plasma incubated with VX (15.11 µg/mL) was treated (*n* = 2) with pronase at 37 °C to analyze biomarker-adducts Cys^34^(-*DPAET*)Pro and Tyr-*EMP* at time points indicated. Data points represent M ± SD of respective peak areas (qualifier ion Qual I, Tables 1, 2) obtained from duplicate proteolysis. *DPAET*: leaving group of VX 2-(diisopropylamino)ethanethiol; *EMP* VX-derived ethyl methylphosphonic acid

To define the optimum duration of proteolysis yielding maximum biomarker concentrations, the time-dependent formation of adducts was monitored. As illustrated in Fig. 5 (filled circles), the concentration of Cys^34^*(-DPAET)*Pro rapidly increased within the first 1.5 h reaching a stable plateau. This plateau indicates the stability of this marker making it a valuable target for verification analysis. The concentration of Tyr*-EMP* (Fig. 5, open circles) also raised rapidly within the first 1.5 h but showed a slower but steady increase within the following 4.5 h. Accordingly, Tyr*-EMP* is also suited as a biomarker from rat plasma not subject to any degradation process. With respect to a reasonable time for the sample preparation workflow, a period of 3 h was fixed for the standard protocol of proteolysis.

### Dose–response analysis and LOI of biomarkers

Over the entire VX concentration range tested in vitro the yield of Cys^34^*(-DPAET)*Pro could be linearly intrapolated in two intervals with differing slopes: a first interval covering the low concentrations ([VX] 1.84 ng/mL–0.94 µg/mL) exhibiting a higher slope (m = 60,085; R^2^ = 0.9985) and a second interval for higher concentrations ([VX] 1.89 µg/mL–7.55 µg/mL) that exhibited a lower slope (m = 38,916, R^2^ = 0.9996) (results not shown). The initial increase of the disulfide-adduct concentration at low VX concentrations was presumably due to the rapid phosphonylation of proteins by the more reactive P( – ) enantiomer of VX (John et al. 2020; Reiter et al. 2008) releasing larger amounts of the leaving group *DPAET*. Accordingly, the second phase indicated that phosphonylation appeared only with either the less reactive P( +) enantiomers of VX or with other less reactive Tyr residues (John et al. 2020; Reiter et al. 2008). Furthermore, increasing VX concentrations might favor an alteration of the 3D structure of albumin due to phosphonylation of multiple different amino acid residues and cracking of disulfide bonds with potential impact on accessibility of the free thiol group on Cys^34^ reducing the yield of Cys^34^*(-DPAET)*Pro.

The LOI of Cys^34^*(-DPAET)*Pro was found at a VX concentration being as low as 11.07 ng/mL (Fig. 2D) still fitting the ion ratio of Qual II/Qual I obtained from a VX reference within the allowed tolerance interval (69.0% ± 13.8%) based on the guidelines of the OPCW (Organisation for the prohibition of chemical weapons 2020b). The LOI of Tyr*-EMP* was found at 1.89 µg/mL VX (ion ratio Qual II/Qual I 80.8% ± 16.2%; Fig. 2E). The much higher LOI for Tyr*-EMP* when compared to Cys^34^*(-DPAET)*Pro was most presumably due to a huge number of parallel reactions with other amino acids e.g., lysine, serine and threonine that reduced the yield of Tyr-adduction (Ding et al. 2008; Fu et al. 2019b).

In the presence of larger amounts of free *DPAET* or its dimer tetraisopropylcystamine (Kranawetvogl et al. 2018a), disulfide-bridged Cys residues in albumin may also undergo adduction forming MetProCys^448^(-*DPAET*) in HSA (Kranawetvogl et al. 2018a) and LeuProCys^448^(-*DPAET*) in RSA as shown in this report. Accordingly, the latter tripeptide showed an LOI of 0.94 µg/mLVX (Fig. 2F) exhibiting an ion ratio of Qual II/Qual I and its allowed tolerance interval of 54.9% ± 11.0%.

### Freeze-and-thaw stability of protein adducts in plasma

No time-dependent degradation was observed for Cys^34^*(-DPAET)*Pro (RSD 10.3%) and Tyr*-EMP* (RSD 3.1%) indicating excellent stability. In contrast, LeuProCys^448^*(-DPAET)* was degraded by 40% after the first cycle. This might be due to an alteration of the conformation of adducted albumin enabling a disulfide exchange once again or imparing pronase-catalyzed liberation of the tripeptide. Since no further degradation was observed during the next three cycles, LeuProCys^448^*(-DPAET)* is still well suited for biomedical verification.

### Stability of biomarkers in the autosampler

All analytes were stable during storage at 15 °C thus proving their suitability even for larger sets of samples (results not shown).

### Adduct stability in plasma at 37 °C

The biomarker peptides Cys^34^*(-DPAET)*Pro, LeuProCys^448^*(-DPAET)* and Tyr*-EMP* were monitored during storage as measures of the respective albumin-adduct stability. Cys^34^*(-DPAET)*Pro reached a stable plateau after 24 h thus documenting a high stability of the disulfide bond produced. Tyr*-EMP* also reached a maximum concentration after 24 h, but was degraded by about 40% within the next 9 d. This degradation might have been due to any enzymatic or non-enzymatic dephosphonylation process or ageing of this phosphonyl-moiety. Ageing refers to the hydrolysis of the ethoxy-group bound to the phosphorus atom (John et al. 2021). Nevertheless, this biomarker was still traceable very well after 10 d documenting sufficient stability of Tyr-*EMP* suited for verification purposes. The concentration of LeuProCys^448^*(-DPAET)* was maximum after 32 h and decreased within the next 6 d to reach a plateau at approximately 50% held until day 10. Due to this low adduct stability, the biomarker tripeptide appeared less suited for verification purposes again.

### Analysis of in vivo samples

**Fig. 6:**
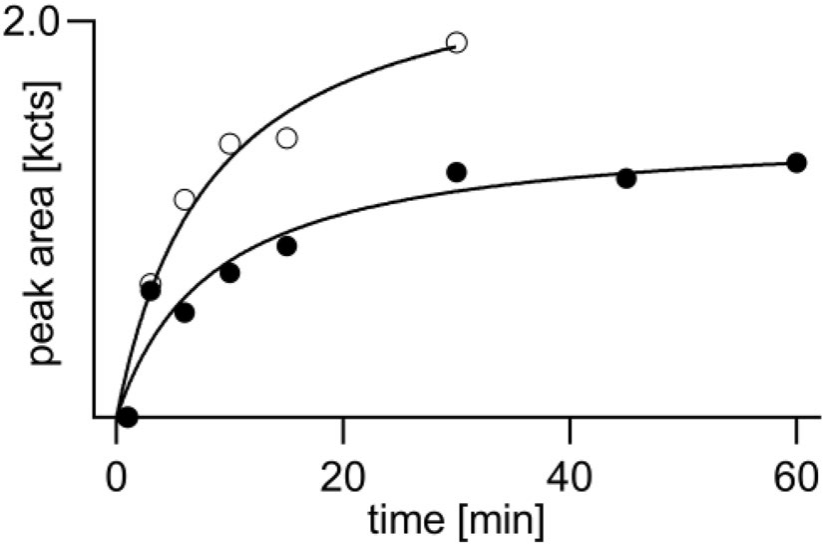
Time-dependent yield of Cys^34^*(-DPAET)*Pro in vivo. Time-dependent increase of Cys^34^*(-DPAET)*Pro in rat plasma after subcutaneous administration of VX (open circles: rat# 1; filled circles: rat# 2). Samples were analysed by µLC-ESI MS/HR MS. *DPAET*: leaving group of VX 2-(diisopropylamino)ethanethiol

In the present study, anaesthetized rats were exposed to VX (s.c.) thus simulating the most likely route of exposure in real case scenarios to elucidate the efficacy of potential novel phosphotriesterase antidotes (Stigler et al. 2022). Absorption of VX via skin happens as a diffusion-dependent process quite slowly (Reiter et al. 2008; van der Schans et al. 2003) causing a concentration-dependent delay of toxic effects (Grob and Harvey 1953; Stigler et al. 2022). Intending to monitor protein adduct formation in vivo plasma samples were analyzed at different times points after exposure. A time-dependent increase of Cys^34^*(-DPAET)*Pro was found during the entire life time of the rats after exposure (Fig. 6, open circles, rat #1, 33 min and filled circles, rat #2, 71 min). This increase underlines the continuous formation of the disulfide-adduct as a consequence of continuous time- and diffusion-dependent VX uptake into the circulating blood. Quality criteria raised by the OPCW concerning the retention time and ion ratios of the analyte in µLC-ESI MS/MS analysis (Organisation for the prohibition of chemical weapons 2020b) were fulfilled in all samples thus documenting the reliability of the method. In addition, blanks taken prior to VX exposure did not show any interference proving the selectivity of the method also suited for in vivo samples.

In contrast, neither Tyr*-EMP* nor LeuProCys^448^(*-DPAET*) were detected over the entire test period in both animals. The lack of detection of these adducts was most presumably due to the low VX dose applied and the resulting low concentrations (nanomolar range) in plasma (Stigler et al. 2022) as well as to the low reactivity of Tyr residues as already observed in vitro (Fig. 4C). Even though the phosphonylated Tyr residue represents a well-established and internationally accepted biomarker of human exposure to OPNA (John et al. 2021) detection in rats failed. Obviously, longer reaction times are required to form adduct amounts sufficient for detection. Our results impressively document the importance of monitoring different primary biomarkers simultaneously for verification purposes.

## Conclusion

The suitability of the biomarker Cys^34^*(-DPAET)*Pro to prove exposure to VX in vivo is documented in this study for the very first time. Moreover, this disulfide-adduct showed superior specifications compared to the well-established Tyr*-EMP* which is also mainly derived from serum albumin. We succeeded in the adaption of the µLC-ESI MS/HRMS (PIS) method to rat plasma, which had been validated before for human plasma and neat HSA only (Kranawetvogl et al. 2016, 2018a). For adaption, we considered the smaller sample volumes obtained from rat (~ 50 µL) and modified the steps of sample preparation. Implementation of two precipitation steps with ACN not only allowed the purification by removal of a majority of matrix components but also resulted in maximum biomarker concentrations in the sample ready for µLC-ESI MS/HR MS (PIS) analysis. In addition, the tripeptide LeuProCys^448^*(-DPAET)* was identified as a novel adduct thus broadening the knowledge of molecular toxicology and interactions of OPNA with endogenous proteins. However, as a sensitive biomarker, it is less suited due to its low concentration. In future studies, the focus will be on the identification of biotransformation products of these adducts to discover new potential biomarkers to verify exposure.

## Abbreviations

AChE: Acetylcholinesterase
ACN: Acetonitrile
BChE: Butyrylcholinesterase
CE: Collision energy
CID: Collision-induced dissociation
CWA: Chemical warfare agents
CWC: Chemical weapons convention
CXE: Carboxylesterase
*d*_*3*_-Atr: Triple deuterated atropine
DPAET: 2-(Diisopropylamino)ethanethiol
EMP: Ethyl methylphosphonic acid
ESI: Electrospray ionization
FA: Formic acid
HSA: Human serum albumin
IS: Internal standard
LC: Liquid chromatography
µLC-ESI MS/HR MS: Microbore liquid chromatography-electrospray ionization high-resolution tandem mass spectrometry
LOI: Limit of identification
M: Mean
[M + H]^+^: Protonated precursor ion
MS: Mass spectrometry
OPCW: Organisation for the Prohibition of Chemical Weapons
OPH: Organophosphorus hydrolase
OPNA: Organophosphorus nerve agents
PIS: Product ion scan
Qual: Qualifier ion
RCF: Relative centrifugal force
RSA: Rat serum albumin
SD: Standard deviation
s.c.: Subcutaneous
VX: *O*-Ethyl-*S*-(2-diisopropylaminoethyl)methylphosphonothioate
